# Three‐Dimensional Digital Visualization System–Assisted Vitrectomy for Infectious Endophthalmitis

**DOI:** 10.1155/joph/3447802

**Published:** 2026-01-28

**Authors:** Lina Guan, Meishuang Li, Wei Fan, Zhengpei Zhang, Yalu Liu, Sujuan Ji, Haiyang Liu, Suyan Li

**Affiliations:** ^1^ Department of Ophthalmology, The Affiliated Xuzhou Municipal Hospital of Xuzhou Medical University, Xuzhou, Jiangsu, China; ^2^ Department of Ophthalmology, Xuzhou First People’s Hospital, Xuzhou, Jiangsu, China; ^3^ Eye Disease Prevention and Treatment Institute of Xuzhou, Xuzhou, Jiangsu, China; ^4^ Xuzhou Medical University, Xuzhou, Jiangsu, China, xzmc.edu.cn

**Keywords:** infectious endophthalmitis, three-dimensional digital visualization system, vitrectomy

## Abstract

**Aim:**

To compare the surgical outcomes and assess the effectiveness of a three‐dimensional digital visualization system (3DVS) versus traditional microscope–assisted pars plana vitrectomy in the management of infectious endophthalmitis.

**Methods:**

A retrospective case series study was conducted on 29 patients diagnosed with infectious endophthalmitis who underwent 23‐gauge transconjunctival vitrectomy between 1 Jan. 2020 and 31 Aug. 2023. Of all these patients, 16 cases underwent vitrectomy‐assisted by the 3DVS (3D group), and the other 13 cases by traditional microscope (eyepiece group). The main comparison focuses on the differences between the two systems in terms of operation time, the brightness of the endoillumination, complications, and preoperative and final best‐corrected visual acuity (BCVA, logMAR).

**Results:**

There were no significant differences in baseline characteristics between the two groups, with trauma being the most prevalent cause of infection (10 vs 8). The positive detection rate of pathogenic bacteria exceeded 40% in both groups (43.75% vs. 46.15%). The results showed that the incidence of complications, including high intraocular pressure (3 vs. 4) and retinal detachment (4 vs. 3), did not differ significantly between the groups (chi‐square = 0.2857, *p* = 0.5930). The mean operation time was slightly shorter in the 3D group (75.94 ± 25.70 min) compared to the eyepiece group (82.31 ± 25.38 min, *p* = 0.5102). However, the 3D group exhibited significantly lower endoillumination (25%–35%) than the eyepiece group (40%–50%, *p* < 0.0001). Both groups demonstrated significant improvement in BCVA at the end of follow‐up (*p* = 0.0006, *t* = 4.321). The mean final BCVA for the 3D group was 1.373 ± 0.9824 logMAR, which was modestly superior to the eyepiece group’s mean of 1.805 ± 0.9549 logMAR.

**Conclusion:**

The 3DVS provides comparable surgical outcomes to the traditional microscope, with the advantages of clearer intraoperative visualization, lower required illumination, and optimized ergonomic design. It is suitable for complex and prolonged endophthalmitis surgery, offering excellent safety and efficacy.

## 1. Introduction

Endophthalmitis, an infectious condition affecting the intraocular fluids, poses a severe threat to vision and can lead to permanent blindness if not promptly and effectively treated [1, 2]. It is characterized by inflammation within the eye, typically caused by bacterial, fungal, or viral pathogens [3–6]. The condition necessitates urgent intervention to preserve vision and even the eyeball, with pars plana vitrectomy combined with intravitreal antibiotics being a widely accepted treatment approach. This procedure aims to remove the infected vitreous, reduce inflammation, and improve the delivery of antibiotics to the site of infection. Despite the established benefits, performing vitrectomy in cases of endophthalmitis presents unique challenges. The clarity and contrast of the fundus are often compromised due to media opacity from inflammation, which complicates the surgical procedure. In addition, the eye’s susceptibility to phototoxicity, particularly in the inflamed state, mandates careful management of intraoperative illumination to prevent further retinal damage. The delicate nature of the intraocular tissues involved also demands precise surgical manipulation to avoid iatrogenic injury.

The advent of the three‐dimensional digital visualization system (3DVS) has revolutionized the field of vitreoretinal surgery by addressing several of these challenges. The system provides a stereoscopic view of the surgical field, enhancing the surgeon’s depth perception and spatial orientation. This improved visualization can facilitate more precise and efficient surgical maneuvers, potentially reducing surgical time and improving patient outcomes. Furthermore, 3DVS offers the advantage of reduced retinal phototoxicity by allowing the surgeon to operate with lower light intensity, as the system’s advanced optics and image processing can compensate for the reduced illumination. The application of 3DVS in vitreoretinal surgery was first introduced by Eckardt and Paulo in 2016 [7], and since then, the system has been increasingly adopted for a variety of surgical indications, including macular surgery, retinal detachment repair, and the management of diabetic eye disease [8]. However, the use of 3DVS in the context of vitrectomy for infectious endophthalmitis has been less explored, with limited reports in the literature. In a preliminary case series published as a preprint, our team initially reported the technical feasibility and favorable outcomes of 3DVS‐assisted vitrectomy in 11 patients with infectious endophthalmitis, demonstrating its safety and potential benefits in this complex scenario [9]. Building upon that foundational experience, the present study aims to provide a more robust evaluation by expanding the sample size, incorporating a traditional microscope control group, and conducting a comparative analysis of surgical parameters and outcomes to quantitatively assess the effectiveness and advantages of the 3DVS system.

In this report, we present a series of cases involving the treatment of endophthalmitis using a 3DVS‐assisted vitrectomy. Our objective is to share our experiences with this technology and to evaluate its effectiveness in the management of endophthalmitis by comparing it with the traditional microscope.

## 2. Participants and Methods

### 2.1. Ethical Approval

The study adhered to the principles of the Declaration of Helsinki and received approval from the Ethics Committee of Xuzhou First People’s Hospital (No. xyyll [2022]‐XJSFX‐124). Written informed consent was obtained from all participants.

### 2.2. Participants

This retrospective case series study included 29 patients diagnosed with infectious endophthalmitis who underwent 23‐gauge transconjunctival vitrectomy using the same Constellation Table‐Top Vision System (Alcon Laboratories, Inc.) by two surgeons between 1 Jan. 2020 and 31 Aug. 2023. These two surgeons have similar qualification and surgical experience, with one specializing in 3DVS surgeries and the other in traditional microscope surgeries. Patient assignment to each surgeon was primarily based on the surgeon’s scheduled availability and the patient’s presentation timing, rather than specific patient or disease characteristics. Of all these patients, 16 cases underwent vitrectomy‐assisted by the NGENUITY 3D Visualization System (Alcon Laboratories, Inc.) (3D group), and the other 13 cases with traditional microscope (Carl Zeiss Microscopy GmbH, Jena, Germany) (eyepiece group).

Patients with infectious endophthalmitis were diagnosed based on a history of ocular trauma, or ocular surgery, endogenous infection, as well as symptoms such as redness, pain, visual decline, photophobia, and tearing in the eye. Slit‐lamp and ocular B‐ultrasound examinations revealed the accumulation of pus in the anterior chamber or vitreous.

### 2.3. Inclusion Criteria

1. Presence of predisposing factors, including ocular trauma, recent ocular surgery, or evidence of endogenous infection.
2. Clinical diagnosis of infectious endophthalmitis, characterized by acute ocular pain and vision loss, accompanied by signs such as conjunctival congestion, hypopyon, or vitreous opacities, with supporting evidence from B‐scan ultrasonography showing findings consistent with vitreous inflammation or debris.

### 2.4. Exclusion Criteria

1. Patients diagnosed with noninfectious uveitis, endophthalmitis, or other sterile inflammatory conditions.
2. Cases lacking complete medical documentation, specifically intraoperative details or essential follow‐up data required for the study.

### 2.5. Surgical Techniques

All patients underwent 23‐gauge transconjunctival pars plana vitrectomy. The 3D group was facilitated by a 3DVS, which utilized a high dynamic range camera that connected to the surgical microscope and transmitted overlapping images to a 3D screen. The surgeon, wearing polarized glasses, viewed a stereoscopic representation of the surgery on the screen. The eyepiece group used the traditional microscope.

Before vitrectomy, if present, traumatic cataract was simultaneously removed, or in cases of postcataract surgery infection with significant opacification or instability, the intraocular lens was extracted. A vitreous specimen was aspirated from the sclerotomy site for bacterial culture. The infusion solution consisted of 500 mL of balanced salt solution with 0.4 mL of epinephrine and 200 mg of cefotaxime sodium added. After core vitrectomy, the peripheral vitreous was thoroughly shaved with scleral depression. The choice of tamponade depended on both the condition of retinal detachment and the changes in the retina and blood vessels. If the exudate on the retinal surface can be blown up with a flute needle, only vitrectomy may be performed. If the retinal vessels were obscured by exudate or even show segmental white sheath–like changes, C_3_F_8_ or filtered air was selected as the tamponade. If the retina was necrotic and dissolved, silicone oil was chosen. At the end of the surgery, intravitreal injections of 1 mg/0.1 mL vancomycin and 2 mg/0.1 mL cefotaxime sodium were administered. The supplemental video file provides a demonstration of the essential steps of the technique procedure.

### 2.6. Follow‐Up

All patients were followed up for a period ranging from 3 to 6 months postoperatively. In cases where combined cataract extraction was performed, secondary intraocular lens implantation was carried out either 3 months after the initial surgery or concurrently with silicone oil removal. For patients with silicone oil–filled eyes, follow‐up continued for at least 3 months after the removal of the silicone oil. During each follow‐up visit, comprehensive evaluations were conducted, including visual acuity assessments, intraocular pressure measurements, and dilated fundus examinations.

### 2.7. Statistical Analysis

We collected data on various parameters, including the duration of the operation, brightness of the endoillumination, cause of infection, tamponade, complications, and the preoperative and final best‐corrected visual acuity (BCVA). To analyze the visual acuity, the decimal values were converted to the logarithm of the minimum angle of resolution (logMAR) using the formula: logMAR = −log (decimal VA) [10]. Counting fingers, hand movement, light perception, and no light perception were assigned logMAR values of 2.1, 2.4, 2.7, and 3.0, respectively [11]. The statistical analysis was performed using the GraphPad Prism 8.0 software. Continuous data were presented as mean ± standard deviation (SD). To compare the BCVA between the two groups, an unpaired *t*‐test was used, while for comparing preoperative and final BCVA, a paired *t*‐test was employed. The chi‐square test was used for comparisons of the following data: gender, cause of infection, positive rate of pathogen detection, tamponade, and complications. A *p* value < 0.05 was considered statistically significant. No formal sample size calculation was performed a priori due to the retrospective nature and the relative rarity of the condition; this is acknowledged as a limitation.

## 3. Results

This study encompassed 29 eyes from 29 patients. Of all these patients, 16 cases underwent vitrectomy‐assisted by the 3DVS (3D group), and the other 13 cases by traditional microscope (eyepiece group). As illustrated in Table 1, the baseline characteristics, including age, gender, preoperative BCVA, positive rate of pathogen detection, cause of infection, and duration of infection, did not exhibit statistically significant differences between the two groups.

**TABLE 1:** Comparison of the baseline data between the two groups.

	**3D group**	**Eyepiece group**		**p** **value**

Age (years)	51.63 ± 15.63	48.23 ± 25.75	*t* = 0.4381	0.6648
Gender				
Male	13 (81.25%)	10 (76.92%)	Chi‐square = 0.082	0.7748
Female	3 (18.75%)	3 (23.08%)		
BCVA (LogMAR)	2.228 ± 0.5786	2.446 ± 0.2066	*t* = 1.293	0.2069
Pathogen detection				
Positive	7 (43.75%)	6 (46.15%)	Chi‐square = 0.017	0.8970
Negative	9 (56.25%)	7 (53.85%)		
Cause of infection				
Traumatic	10 (62.5%)	8 (61.54%)	Chi‐square = 0.922	0.6307
Postoperative	5 (31.25%)	5 (38.46%)		
Endogenous	1 (6.25%)	0 (0%)		
Duration of infection (days)	6.458 ± 5.783	5.692 ± 7.631	*t* = 0.3077	0.7607

The age of patients in the 3D group is 51.63 ± 15.63, while in the eyepiece group, it is 48.23 ± 25.75, with no significant difference (*p* = 0.6648). Among the 16 patients in the 3D group, there are 13 males and 3 females. Out of the 13 patients in the eyepiece group, there are 10 males and 3 females, which has no significant difference (*p* = 0.7748). The preoperative BCVA of patients in the 3D group is 2.228 ± 0.5786, while in the eyepiece group, it is 2.446 ± 0.2066, also with no significant difference (*p* = 0.2069). Notably, the positive detection rate of pathogenic bacteria surpassed 40% in both groups. The positive rate of pathogen detection of patients in the 3D group is 43.75% (7 out of 16 patients), while in the eyepiece group, it is 46.15% (6 out of 13 patients), with no significant difference (*p* = 0.8970). Trauma emerged as the most prevalent cause of infection, accounting for 18 cases out of 29 cases. In the 3D group, there are 10 cases of trauma (62.5%), while in the eyepiece group, there are 8 cases (61.54%).

**FIGURE 1:**
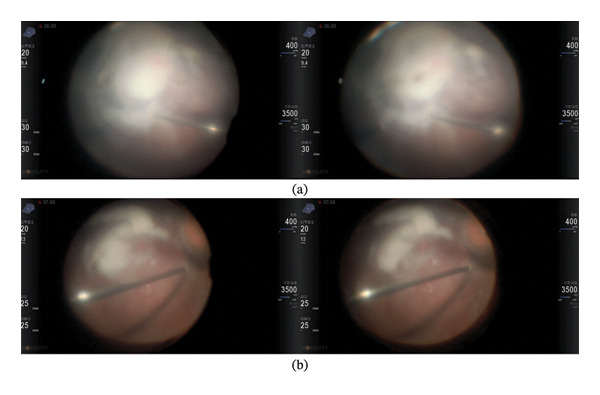
Intraocular illumination was adjusted to the lowest intensity sufficient for clear visualization. (a) The initial brightness was 30%. (b) With the surgical field becoming progressively clearer as the procedure advances, the brightness was reduced to 25%.

Table 2 presents the surgical details and treatment outcomes for both groups. The mean operation time for the 3D group was 75.94 ± 25.70 min, slightly shorter than the 82.31 ± 25.38 min recorded for the eyepiece group (*p* = 0.5102). Intraoperative illumination was adjusted to the minimal level required for vitrectomy in both groups (Figure 1). Notably, the brightness in the 3D group was significantly lower, ranging between 25% and 35% of maximum luminance (*t* = 11.04, *p* < 0.0001), compared to 40%–50% in the eyepiece group.

**TABLE 2:** Comparison of the outcomes between the two groups.

	**3D group**	**Eyepiece group**		**p** **value**

Operation time (min)	75.94 ± 25.70	82.31 ± 25.38	*t* = 0.6674	0.5102
Endoillumination (%)	29.06 ± 3.276	44.62 ± 4.312	*t* = 11.04	< 0.0001
Tamponade				
Silicone oil	7 (43.75%)	5 (38.46%)	Chi‐square = 0.101	0.9508
Filtered air	2 (12.5%)	2 (15.38%)		
Balanced salt solution	7 (43.75%)	6 (46.15%)		
Complications				
Retinal detachment	4 (25.00%)	3 (23.08%)	Chi‐square = 0.2857	0.5930
Elevated intraocular pressure	3 (18.75%)	4 (30.77%)		
Follow‐up (months)	6.063 ± 2.695	5.615 ± 2.663	*t* = 0.4467	0.6587
BCVA (LogMAR)				
Preoperation	2.228 ± 0.5786	2.446 ± 0.2066	*t* = 1.293	0.2069
Follow‐up	1.373 ± 0.9824	1.805 ± 0.9549	*t* = 1.193	0.2433
Paired *t*‐test	*t* = 4.321	*t* = 2.617		
*p* value	*p* = 0.0006	*p* = 0.0225		

No statistically significant differences were observed between the two groups in terms of tamponade use (chi‐square = 0.101, *p* = 0.9508), complication rates (chi‐square = 0.2857, *p* = 0.5930), and follow‐up time (*t* = 0.4467, *p* = 0.6587). Both groups reported 7 postoperative complications. Specifically, in the 3D group, 3 patients (18.75%) experienced elevated intraocular pressure postoperatively, compared to 4 patients (30.77%) in the eyepiece group. However, intraocular pressure returned to normal levels following medication in both groups. Regarding retinal detachment, four patients (25.00%) in the 3D group developed this condition, with one undergoing a second surgery for retinal reattachment and three retaining silicone oil due to poor vision. In the eyepiece group, three patients (23.08%) experienced retinal detachment. Among them, one underwent a second surgery for retinal reattachment, one became silicone oil–dependent, and one underwent enucleation due to uncontrollable infection.

Both groups showed substantial improvements in BCVA by the end of the follow‐up period. The magnitude of BCVA improvement was statistically significant in both groups, with the 3D group demonstrating a preoperative to follow‐up difference with *p* = 0.0006 and *t* = 4.321, and the eyepiece group showing a difference with *p* = 0.0225 and *t* = 2.617. The mean final BCVA for the 3D group was 1.373 ± 0.9824 logMAR, modestly better than the eyepiece group’s mean of 1.805 ± 0.9549 logMAR.

## 4. Discussion

The surgical outcomes and the effectiveness of 3DVS in pars plana vitrectomy in the management of infectious endophthalmitis were presented. In both groups of patients (3D group vs. eyepiece group), there was no statistically significant difference in baseline characteristics (i.e., age, gender, preoperative BCVA, positive rate of pathogen detection, cause of infection, and duration of infection), ensuring the reliability of the results comparison. The comparison of the outcomes between the two groups is very clear (Table 2). All cases in the 3D group successfully underwent vitrectomy, with no need to switch to traditional microscopy or terminate the surgery prematurely. The endoilluminator’s brightness was fine‐tuned to the minimal level necessary for the procedure, ranging between 25% and 35%, markedly lower than the eyepiece group, which required approximately 40%–50% light intensity. In addition, the camera gain was electronically amplified to a setting of three, with the camera’s iris diaphragm fully dilated at 100%. Notably, all surgeries were conducted without the use of color filters. The surgeon observed that these settings enhanced visualization within the murky refractive medium of endophthalmitis. Furthermore, intraoperative visibility progressively improved as the surgery advanced. The digital fusion technology facilitated the display of these real‐time surgical parameters on the screen, aiding in precise monitoring and offering educational benefits.

Our findings demonstrate that the implementation of the 3DVS offers comparable surgical outcomes to the traditional microscope for endophthalmitis surgery. Real‐time digital signal processing enhances the brightness and clarity of images, providing superior intraoperative visualization and a broader field of view, which is ideal for such intricate procedures. The high‐definition of the 3DVS ensures excellent visualization even in gas‐filled eyes, enabling us to omit the use of perfluorocarbon liquid (PFCL) in the treatment of rhegmatogenous retinal detachment [12]. Other similar studies, such as those by Eckardt [1], have shown that while electronically boosting the camera’s signal gain enhances image brightness, it also introduces more digital noise. Notably, a combination of 20% light intensity with a Gain 3 setting achieves the same image brightness as 80% light intensity with a Gain 1 setting, effectively quadrupling the light source intensity without signal amplification. There is a consensus that the 3DVS requires lower illumination compared to traditional microscopes, whether used for anterior or posterior segment surgeries [13, 14]. Leveraging its superior brightness, visualization of the peripheral vitreous becomes feasible through scleral transillumination [13]. Todorich et al. [15] employed this scleral transillumination technique during vitrectomy for common vitreoretinal diagnoses. Therefore, compared to conventional microscopes, the 3DVS provides brighter images at lower light intensities. While this study did not quantitatively assess retinal phototoxicity, the significantly lower intraocular illumination used in the 3D group is theoretically beneficial in reducing the risk of phototoxic retinal damage, a consideration particularly important in inflamed endophthalmitis eyes. Supporting this notion, a recent randomized controlled trial by our team demonstrated that the 3D head‐up system significantly reduced the light intensity reaching the operator’s ocular surface and caused less impact on tear secretion and subjective discomfort compared to the traditional microscope eyepiece system during simulated vitrectomy [16].

Moreover, the 3DVS alleviates neck discomfort and visual fatigue for ophthalmologists to some extent. Studies on musculoskeletal disorders among ophthalmologists reveal a strong correlation between occupation‐related musculoskeletal pain, particularly in the neck, and the duration of surgical procedures [17]. Especially when using traditional microscopes, surgeons are forced to maintain a prolonged head‐down posture while observing through the eyepiece, often leading to neck discomfort and visual fatigue. The 3DVS addresses this issue by allowing surgeons to lift their heads, thereby reducing musculoskeletal strain [14]. In addition, the system enables operations at higher magnifications on larger display screens, providing equal ease of viewing all areas of the surgical field, which effectively minimizes visual fatigue. A study comparing the effects of the two surgical systems on the ocular surface of operators demonstrated that the 3DVS group experienced greater subjective comfort and had less impact on nonintrusion tear meniscus height (NIKTMH), bulbar redness, and strip meniscometry tube (SMTube) measurements [16]. Furthermore, it allows the entire surgical team to observe the procedure with the same high‐definition clarity [18]. When integrated with digital fusion technology, which displays surgical parameters in real‐time on the screen, it significantly enhances the educational value of the procedure.

Beyond visual appeal, ergonomic design, and comparable efficacy and safety to traditional microscopes [17], the 3DVS neither prolongs operation time nor limits its applicability, in contrast to traditional microscopes, being suitable for a broad spectrum of vitreoretinal diseases and intricate surgical procedures. For example, we have found that the benefits of the 3DVS can be extended to complex endophthalmitis vitrectomies. Intraoperatively, the illumination was set at approximately 25%–30% of the maximum luminance, markedly lower than that required for conventional microscopy. This reduction in intraocular illumination intensity is a notable technical advantage of the 3D system.

Some surgeons may encounter challenges when shifting from conventional microsurgery to 3DVS surgery, experiencing a spectrum of discomfort collectively termed as “3D asthenopia.” Factors such as exotropia and conflicting vergence‐accommodation can exacerbate these symptoms [19]. It is worth noting that the “comfort zone” can differ based on individual circumstances and preferences [20, 21]. Numerous respondents highlighted anterior segment surgeries as the primary obstacle [13], potentially due to excessive zoom or a 90‐millisecond delay. However, for the majority, the learning process was short, with many feeling highly comfortable and proficient after just three or four surgeries [18]. Generally, a 4‐week training period suffices. Once accustomed, surgeons favor the 3DVS system over traditional microscopy because of its superior visual clarity and enhanced comfort [13, 22, 23]. A surgeon questionnaire survey revealed that 3D technology scored higher in image resolution, field of view, ergonomics, and technical feasibility compared to traditional microscopes [13].

Due to the rarity of endophthalmitis, this study only covered a small number of cases. Furthermore, as a retrospective analysis, although we have identified a decrease in intraoperative illumination intensity in the 3D group, we lack quantitative indicators to evaluate retinal phototoxic injury. In future research, we will focus on the quantitative relationship between 3DVS and retinal phototoxic injury.

This study has several limitations. First, its retrospective nature and the involvement of two surgeons, each dedicated to one visualization system, introduce potential for operator bias, although their experience levels were comparable. Patient assignment was based on scheduling logistics rather than randomization. Second, the sample size is relatively small, and no formal power calculation was performed, which may limit the generalizability of the findings and the ability to detect smaller differences between groups. Third, as a retrospective analysis, although we have identified a significant decrease in intraoperative illumination intensity in the 3D group, we lack quantitative indicators to directly evaluate retinal phototoxic injury in these complex postinfectious eyes. Future prospective studies with larger cohorts and dedicated phototoxicity assessments are warranted. Due to the rarity of endophthalmitis, this study only covered a small number of cases. Furthermore, while lens or IOL removal was performed when deemed necessary for visualization or infection control, the specific impact of phakic status or significant cataract on the relative performance of the two visualization systems was not separately analyzed due to sample size constraints.

## 5. Conclusion

The 3DVS provides surgical outcomes comparable to the traditional microscope in the management of infectious endophthalmitis. It offers enhanced intraoperative clarity, significantly lower required illumination, and an optimized ergonomic design, making it an ideal choice for a wide range of vitreoretinal surgeries, characterized by its exceptional safety and effectiveness. Especially in complex and prolonged endophthalmitis surgeries, this system has proven to be particularly advantageous. Further prospective studies are needed to confirm these findings and quantitatively assess the benefits regarding phototoxicity.

## Author Contributions

Conceptualization: Suyan Li and Haiyang Liu.

Surgery (3DVS group): Lina Guan.

Surgery (traditional microscope group): Wei Fan.

Data curation and formal analysis: Meishuang Li and Yalu Liu.

Investigation and resources: Zhengpei Zhang and Sujuan Ji.

Writing–original draft: Lina Guan.

Writing–review and editing: All authors.

Supervision and project administration: Suyan Li and Haiyang Liu.

## Funding

This work was supported by the Xuzhou Science and Technology Program (CN) (Grant no. KC23164).

## Disclosure

All authors have read and agreed to the published version of the manuscript. The authors acknowledge that a preliminary version of this work was previously published as a preprint on Research Square: Three‐Dimensional Digital Visualization System–Assisted Vitrectomy for Infectious Endophthalmitis, available at: https://www.researchsquare.com/article/rs-2076616/v1.

## Conflicts of Interest

The authors declare no conflicts of interest.

## Data Availability

The data that support the findings of this study are available from the corresponding author upon reasonable request.
